# Supplementary material for: In vitro induction of patterned branchial arch-like aggregate from human pluripotent stem cells
Source: Nat Commun. 2024 Feb 14;15:1351. doi: 10.1038/s41467-024-45285-0 (PMC10867012; doi:10.1038/s41467-024-45285-0)
Supplement: Supplementary file 1 — Supplementary Information [file 41467_2024_45285_MOESM1_ESM.pdf]

Supplementary Information for

In vitro induction of patterned branchial arch-like aggregate from human pluripotent stem cells

Seto *et al.*

Correspondence to:
eiraku@infront.kyoto-u.ac.jp

This PDF file includes:

・Supplementary Figs. 1 to 17

・Supplementary tables 1 to 4

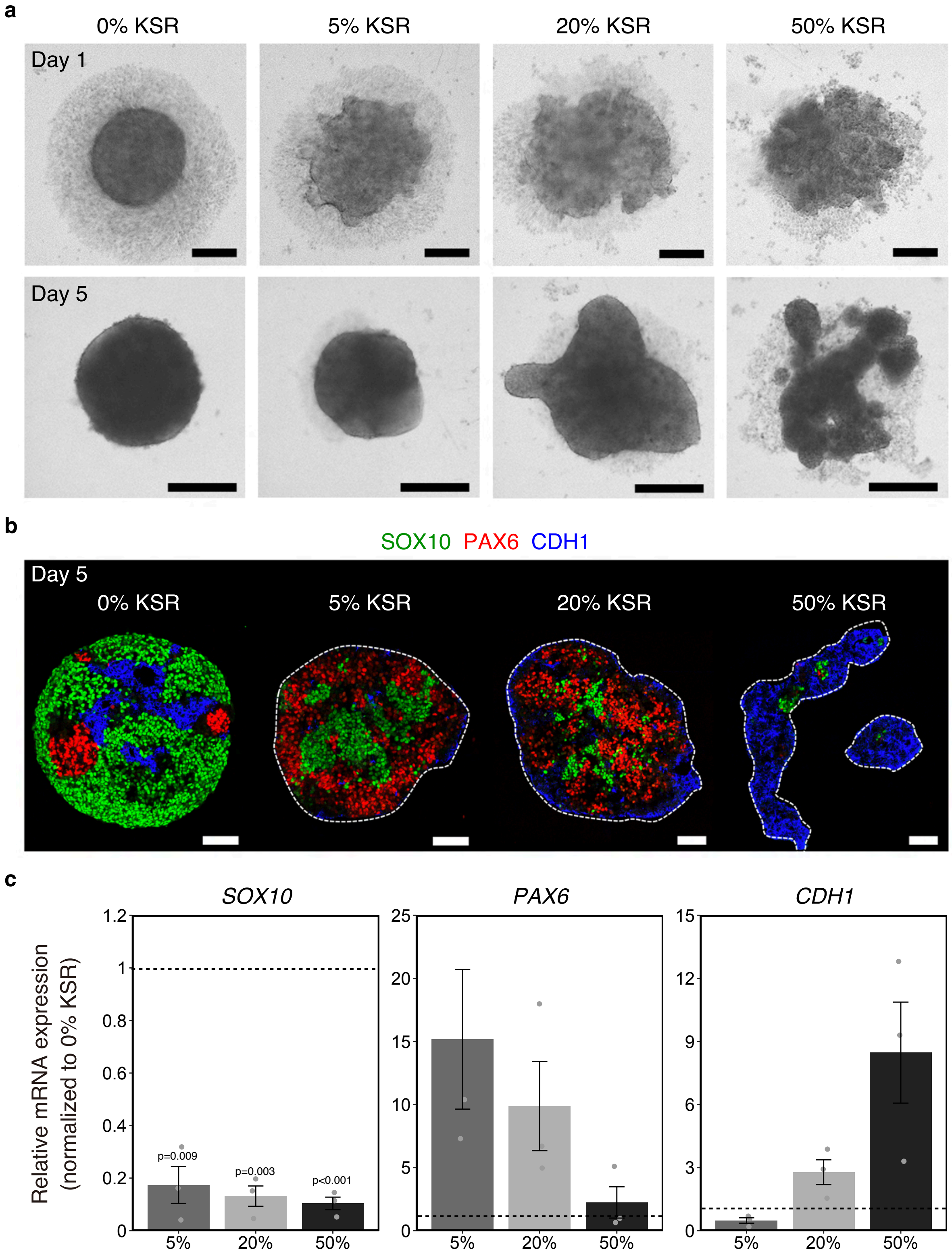

a
0% KSR
5% KSR
20% KSR
50% KSR
Day 1
Day 5
b
SOX10 PAX6 CDH1
Day 5
0% KSR
5% KSR
20% KSR
50% KSR
c
SOX10
PAX6
CDH1
Relative mRNA expression (normalized to 0% KSR)
p=0.009
p=0.003
p<0.001
5%
20%
50%

Supplementary Figure 1. Effect of KSR on NCC-like cell induction

(a) NCC (neural crest cell) induction in NCC_{ind} medium containing various amount of KSR (KnockOut Serum Replacement). The aggregates showed distorted shapes in the presence of KSR. Scale bars, 200 µm (Day 1), 400 µm (Day 5). Three independent cultures were used for experiment and representative images are shown. (b) Immunostaining of day-5 aggregates with antibodies for SOX10 (green), PAX6 (red), and CDH1 (blue). $SOX10^+$ cells were reduced in the aggregates cultured in the presence of KSR while $PAX6^+$ cells and $CDH1^+$ cells increased according to the concentration of KSR. Scale bars, 100 µm. Three independent cultures were used for experiment and representative images are shown. (c) Real-time PCR analysis of SOX10, PAX6, and CDH1. Data are presented as mean ± SEM (n = 3 independent experiments). Two-tailed Student's t-test was used. Source data are provided as a Source Data file.

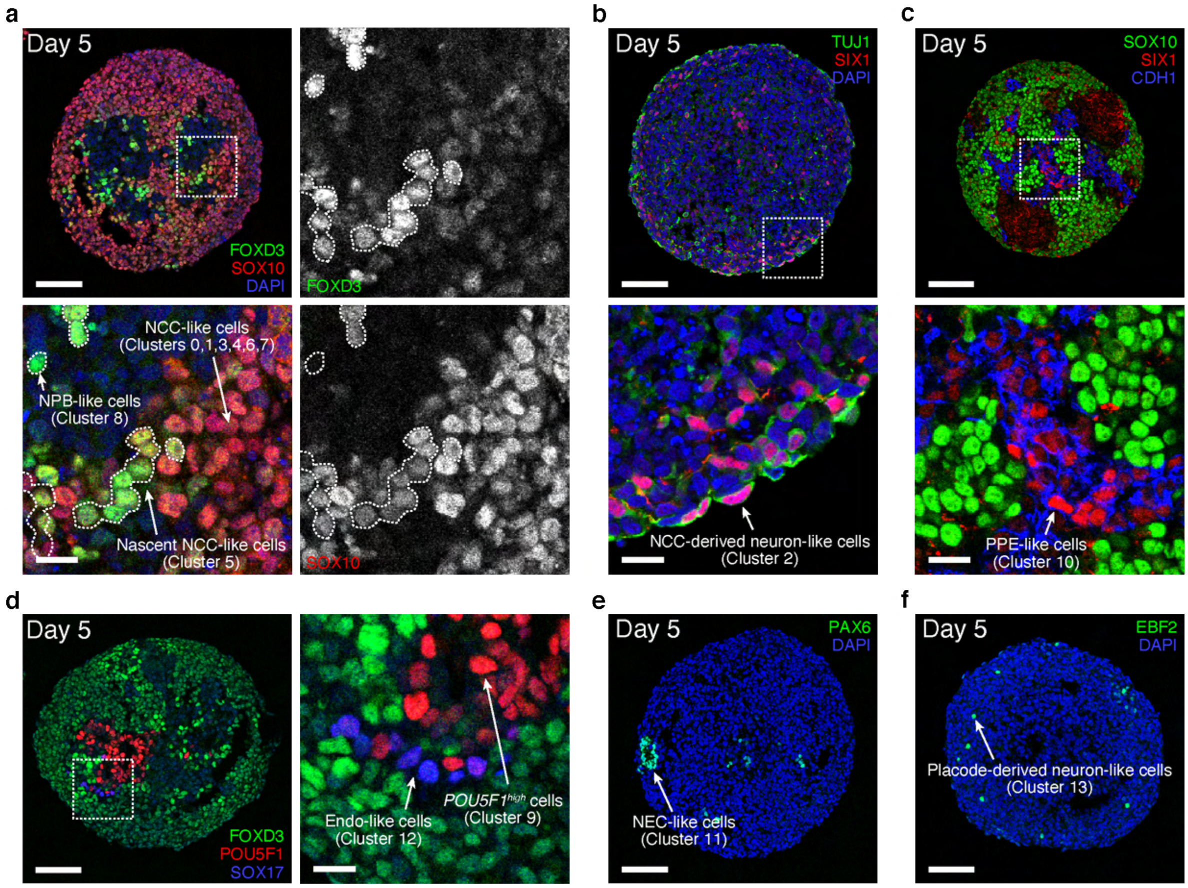

Supplementary Figure 2. Immunohistochemical confirmation of the presence of cell types identified by scRNA-seq at day 5.

(a) Immunostaining of day-5 aggregates with antibodies for FOXD3 (green) and SOX10 (red) identified NCC-like, nascent NCC-like and NPB-like cells. Scale bars, 100 µm (upper left), 20 µm (bottom left). NCC: neural crest cell, NPB: neural plate border. (b) Immunostaining of TUJ1 (green) and SIX1 (red) identified NCC-derived neuron-like cells. Scale bars, 100 µm (upper), 20 µm (bottom) (c) Immunostaining of SIX1 (red) and CDH1 (blue) identified PPE-like cells. Scale bars, 100 µm (upper), 20 µm (bottom). PPE: pre-placodal epithelium. (d) Immunostaining of POU5F1 (red) and SOX17 (blue) identified *POU5F1*^{high} cells and endo-like cells. Scale bars, 100 µm (left), 20 µm (right). Endo: endoderm. (e) Immunostaining of PAX6 (green) identified NEC-like cells. Scale bar, 100 µm. NEC: neuroepithelial cell. (f) Immunostaining of EBF2 (green) identified placode-derived neuron-like cells. Scale bar, 100 µm. Three independent cultures were used for experiment and representative images are shown for each figure.

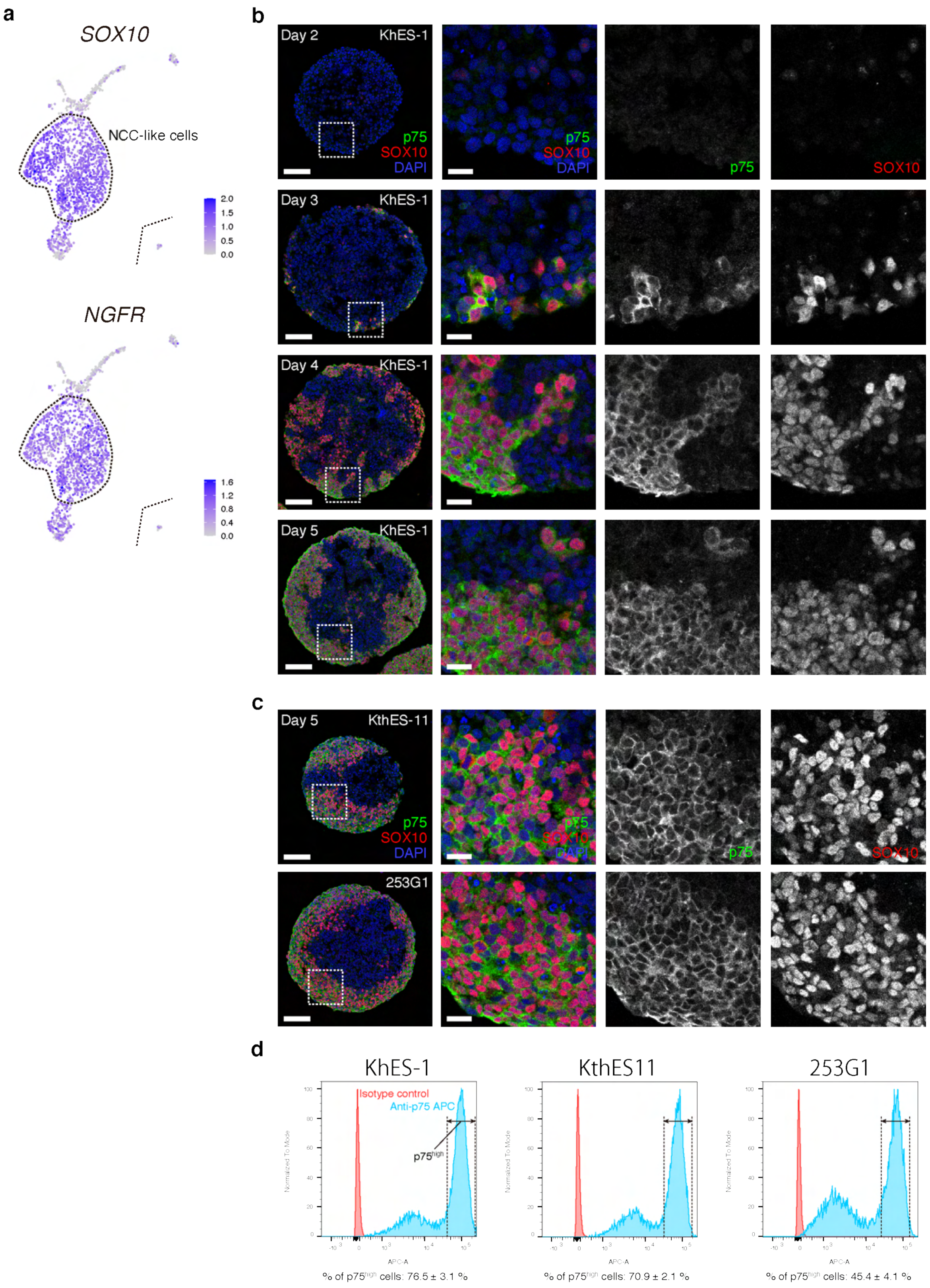
a
SOX10
NCC-like cells
NGFR
b
Day 2
KhES-1
Day 3
Day 4
Day 5
p75
SOX10
DAPI
c
KthES-11
253G1
d
KhES-1
KthES11
253G1
Isotype control
Anti-p75 APC
p75high
% of p75high cells: 76.5 ± 3.1 %
% of p75high cells: 70.9 ± 2.1 %
% of p75high cells: 45.4 ± 4.1 %

Supplementary Figure 3. Characterization of p75 expression at day 5.

(a) Feature plots of day-5 aggregates showing the expression of *SOX10* and *NGFR* (p75) in NCC-like cells (indicated by dotted line). NCC: neural crest cell. (b) Immunostaining of p75 (green) and SOX10 (red) from day2 to day 5 in KhES-1-derived aggregates suggested specific expression of p75 in $SOX10^+$ cells. Scale bars, 100 µm (left), 20 µm (right). Three independent cultures were used for experiment and representative images are shown. (c) Immunostaining of KthES11-derived and 253G1-derived aggregates for p75 (green) and SOX10 (red) also confirmed the expression of p75 in $SOX10^+$ cells. Scale bars, 100 µm. Three and five independent cultures were used for experiment for KthES11 and 253G1, respectively, and representative images are shown. (d) Representative image of flow cytometry analysis of the day-5 aggregates derived from KhES-1, KthES11 and 253G1. The averages of percentage of $p75^{high}$ cells are written below the plot for each cell line tested (n = 3 for KhES-1 and KthES11, n = 5 for 253G1). Source data are provided as a Source Data file. APC: allophycocyanin.

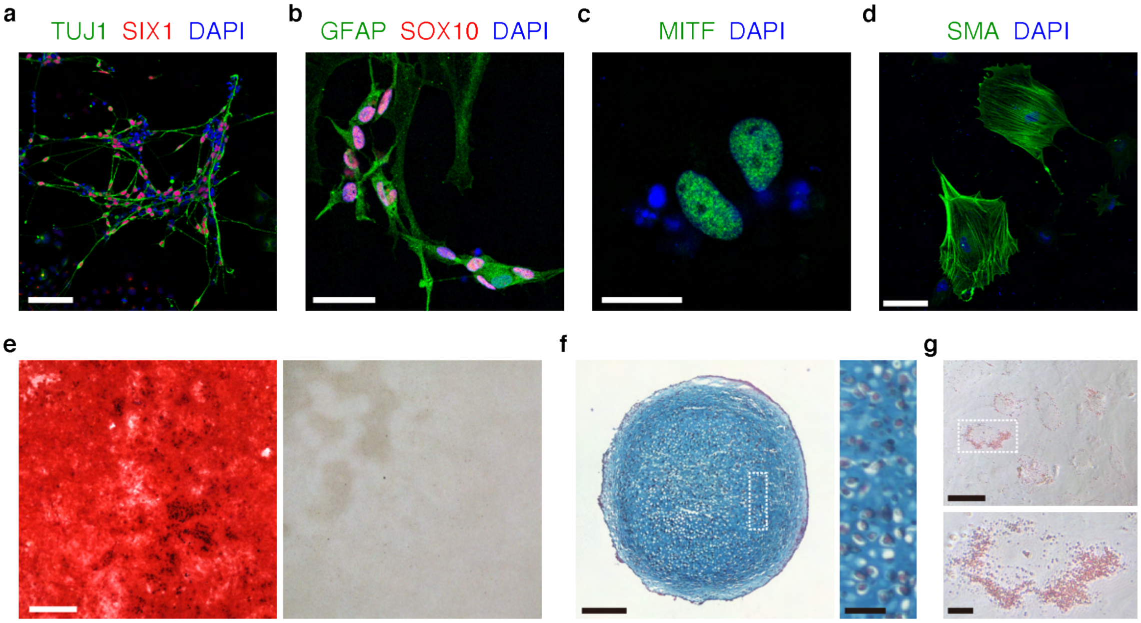

Supplementary Figure 4. Differentiation potential of induced NCC-like cells.

(a) Immunostaining of TUJ1 (green) and SIX1 (red) suggested neuronal differentiation. Cells were cultured on iMatrix-coated dish with NCC_{ind} medium for 7 days. Scale bar, 100 µm (b) Immunostaining of GFAP (green) and SOX10 (red) suggested differentiation into glial lineage. Cells were cultured on iMatrix-coated dish with NCC_{ind} medium for 21 days. Scale bar, 100 µm (c) Immunostaining of MITF (green) suggested differentiation into melanocyte. Cells were cultured on iMatrix-coated dish with NCC_{ind} medium for 7 days. Scale bar, 25 µm (d) Immunostaining of SMA (green) suggested differentiation into smooth muscle cell. Cells were cultured on Geltrex-coated dish with DMEM/F-12 with 10% FBS for 7 days. Scale bar, 100 µm (e) Alizarin Red staining suggested differentiation into osteogenic lineage (left). Cells were cultured under osteogenic condition for 22 days. Control sample cultured with bFGF-added NCC_{ind} medium did not exhibit red staining (right). Scale bar, 500 µm (f) Alcian Blue staining of aggregates cultured under chondrogenic condition for 30 days suggested differentiation into Chondrogenic lineage. Scale bars, 250 µm (left), 50 µm (right) (g) Oil Red O staining of cell cultured under adipogenic condition for 30 days. Scale bars, 100 µm (upper), 20 µm (bottom). Three independent cultures were used for experiment and representative images are shown for each figure.

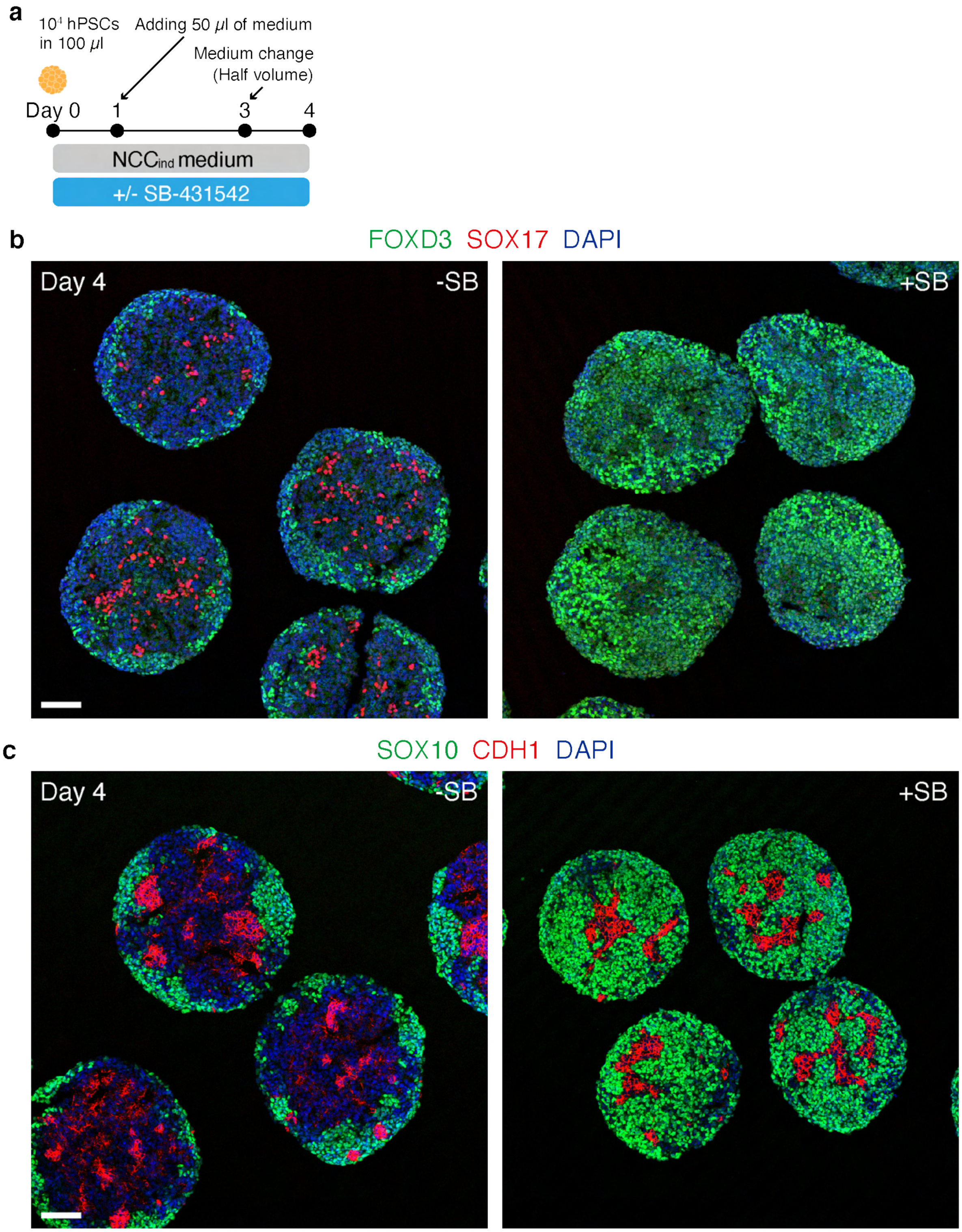
a
10^4 hPSCs in 100 μl
Adding 50 μl of medium
Medium change (Half volume)
Day 0
1
3
4
NCCind medium
+/- SB-431542
b
FOXD3 SOX17 DAPI
Day 4
-SB
+SB
c
SOX10 CDH1 DAPI
Day 4
-SB
+SB

Supplementary Figure 5. Effect of SB-431542 on NCC (neural crest cell)-like cell induction.

(a) Scheme of experiment. SB-431542 was added from day 0 and the aggregates were analyzed at day 4. hPSCs: human pluripotent stem cells. (b) Immunostaining of FOXD3 (green) and SOX17 (red) suggested increase of $FOXD3^{+}$ NCC-like cells and inhibition of $SOX17^{+}$ endoderm-like cell induction in SB (SB-431542)-treated aggregates. Scale bar, 100 μm (c) Immunostaining of SOX10 (green) and CDH1 (red) also suggested increase of NCC-like cells. $CDH1^{+}$ cells (presumptive non-neural ectoderm/pre-placodal epithelium-like cells) did not seem to be affected. Scale bar, 100 μm. Three independent cultures were used for experiment and representative images are shown for each figure.

(

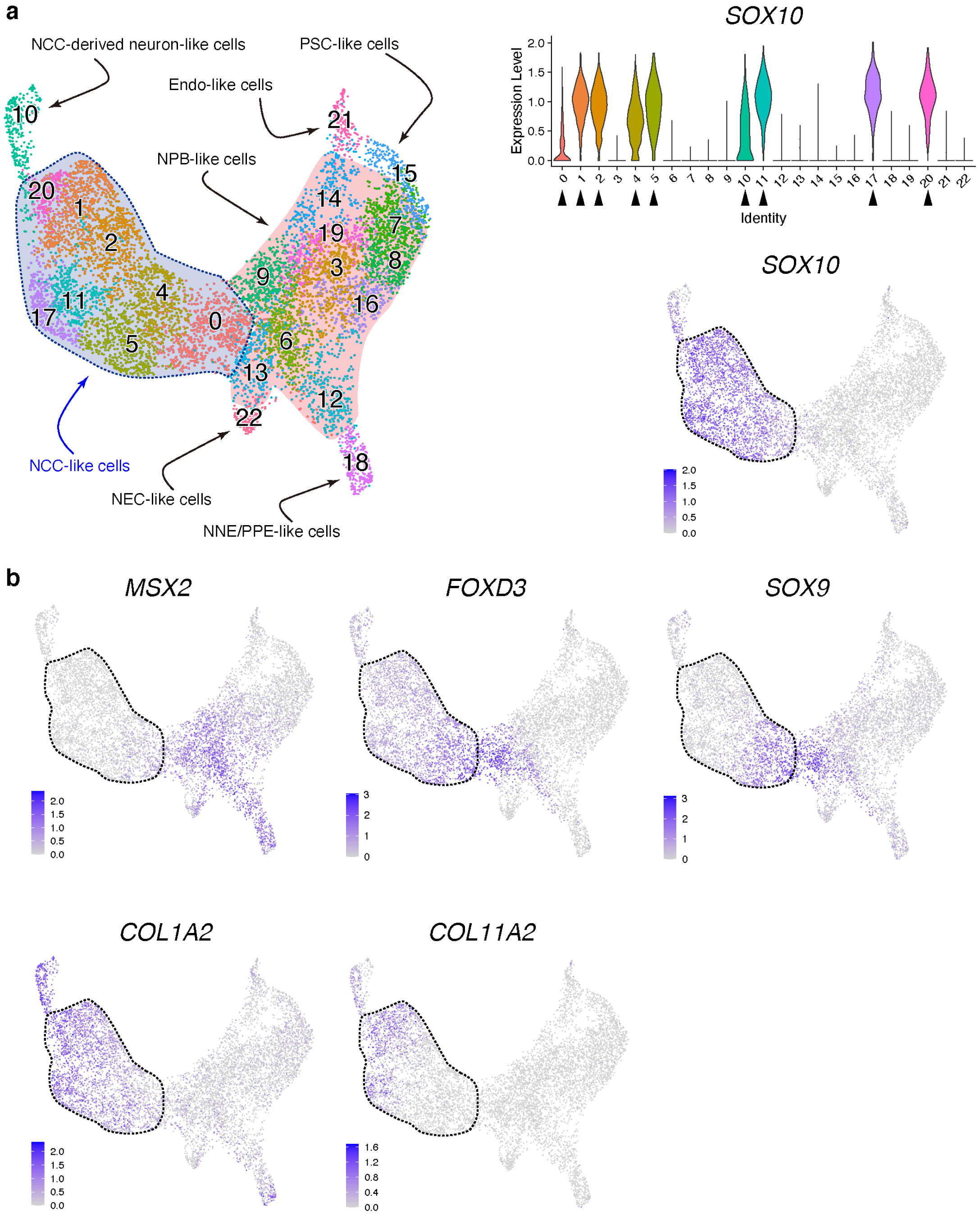
a
NCC-derived neuron-like cells
PSC-like cells
Endo-like cells
NPB-like cells
NCC-like cells
NEC-like cells
NNE/PPE-like cells
SOX10
Expression Level
Identity
b
MSX2
FOXD3
SOX9
COL1A2
COL11A2

Supplementary Figure 6. A variety of state of NCC (neural crest cell)-like cells.

(a) Uniform manifold approximation and projection of merged single cell RNA-seq data (left). Violin plot and feature plots of *SOX10* suggested clusters 0, 1, 2, 4, 5, 11, 17, 20 were NCC-like cells (right). Endo: endoderm, NEC: neuroepithelial cell, NNE/PPE: non-neural ectoderm/pre-placodal epithelium, NPB: neural plate border, PSC: pluripotent stem cell. (b) Feature plot of representative genes expressed differentially in NCC-like cell clusters. *MSX2*, *FOXD3*, and *SOX9* were expressed from NPB-like state to the early phase of NCC-like differentiation. The expression of *MSX2* ceased at very early timing during NCC-like differentiation while the expression of *SOX9* sustained until a little later. *FOXD3* expression seemed to last more later although the expression level became very low. The expression of *COL1A2* was started a little after the differentiation into NCC-like state. The expression of *COL11A2* was started from more later timing. Those data indicated a variety in the cell state of NCC-like cells.

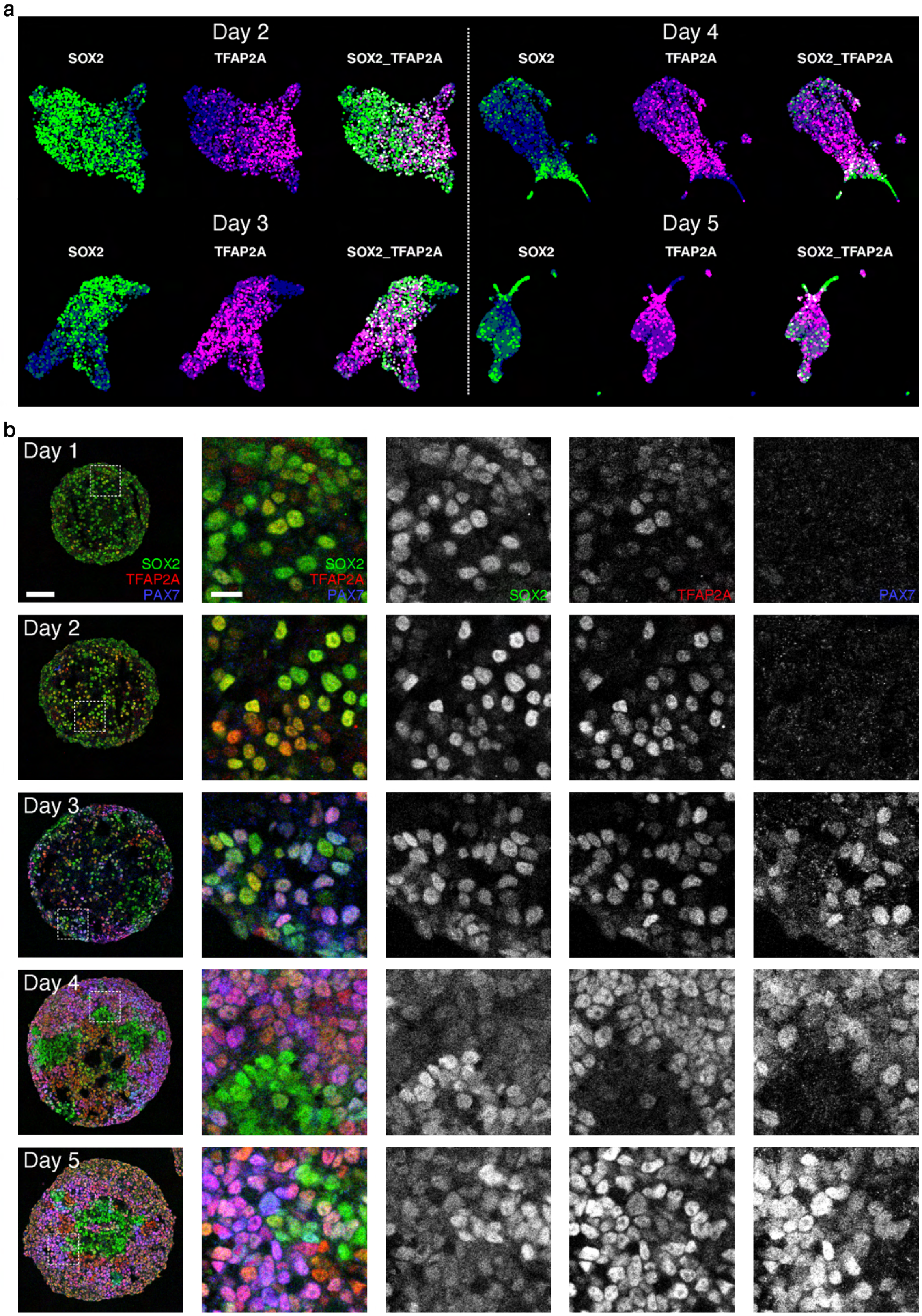
a
Day 2
SOX2
TFAP2A
SOX2_TFAP2A
Day 4
SOX2
TFAP2A
SOX2_TFAP2A
Day 3
SOX2
TFAP2A
SOX2_TFAP2A
Day 5
SOX2
TFAP2A
SOX2_TFAP2A
b
Day 1
SOX2
TFAP2A
PAX7
SOX2
TFAP2A
PAX7
SOX2
TFAP2A
PAX7
Day 2
Day 3
Day 4
Day 5

Supplementary Figure 7. Expression of SOX2 and TFAP2A in the aggregates.

(a) Feature plots of *SOX2* and *TFAP2A* indicated co-expression of them during the differentiation of the aggregates. (b) Immunostaining of SOX2 (green), TFAP2A (red) and PAX7 (blue) revealed co-expression of SOX2 and TFAP2A from day 1 to day 5 although the expression of SOX2 became weak in TFAP2A^{+} cells from day 4. Prior to such downregulation of SOX2, PAX7 came to expressed in subsets of SOX2^{+}/TFAP2A^{+} cells at day3. Then, SOX2^{high} cells were segregated from TFAP2A^{high} cells which expressed PAX7 from day 4 suggesting lineage bifurcation among neural plate border-like cells. Scale bars, 100 μm (left), 20 μm (right). Three independent cultures were used for experiment and representative images are shown for each figure.

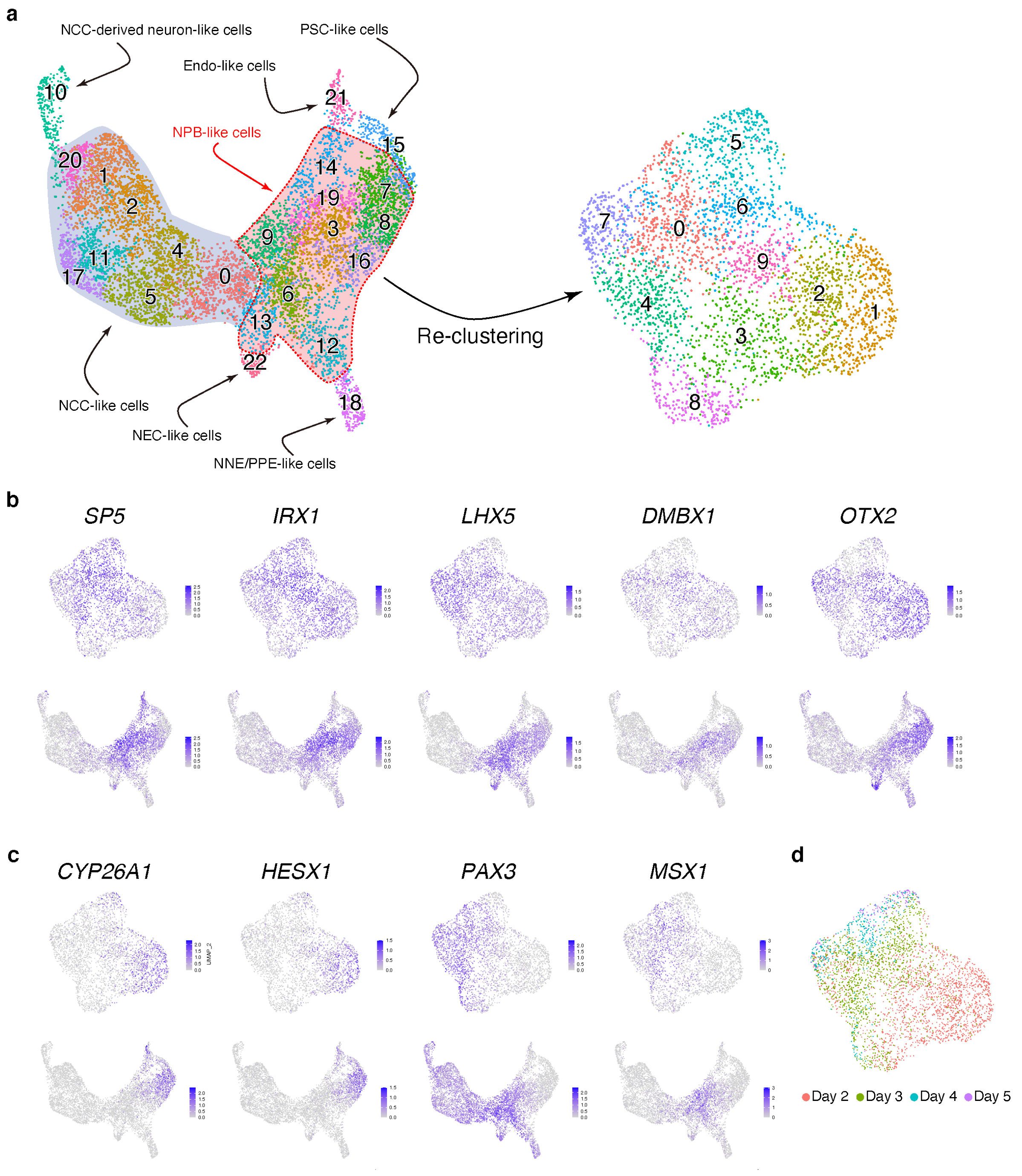
a
NCC-derived neuron-like cells
PSC-like cells
Endo-like cells
NPB-like cells
Re-clustering
NCC-like cells
NEC-like cells
NNE/PPE-like cells
b
SP5
IRX1
LHX5
DMBX1
OTX2
c
CYP26A1
HESX1
PAX3
MSX1
d
Day 2
Day 3
Day 4
Day 5

Supplementary Figure 8. Re-clustering of NPB (neural plate border)-like cell clusters.

(a) Uniform manifold approximation and projection (UMAP) of merged scRNA-seq data (left). NPB-like cells were re-clustered to find marker genes (right). Endo: endoderm, NCC: neural crest cell, NEC: neuroepithelial cell, NNE/PPE: non-neural ectoderm/pre-placodal epithelium, PSC: pluripotent stem cell. (b) Feature plots of *SP5*, *IRX1*, *LHX5*, *DMBX1*, and *OTX2*. These genes were expressed broadly in NPB-like cell clusters. *LHX5*, *DMBX1* and *OTX2* was cranial NCC markers. Upper: re-clustered NPB-like cell clusters, bottom: original data before subsetting. (c) Feature plots of *CYP26A1*, *HESX1*, *PAX3*, and *MSX1*. The expression of *CYP26A1* and *HESX1* did not seem to be overlapped with that of *PAX3* and *MSX1*. *CYP26A1* and *HESX1* were expressed earlier and the others were expressed later during the differentiation as suggested by UMAP in (d). (d) UMAP of re-clustered NPB-like cells colored by their sampling date.

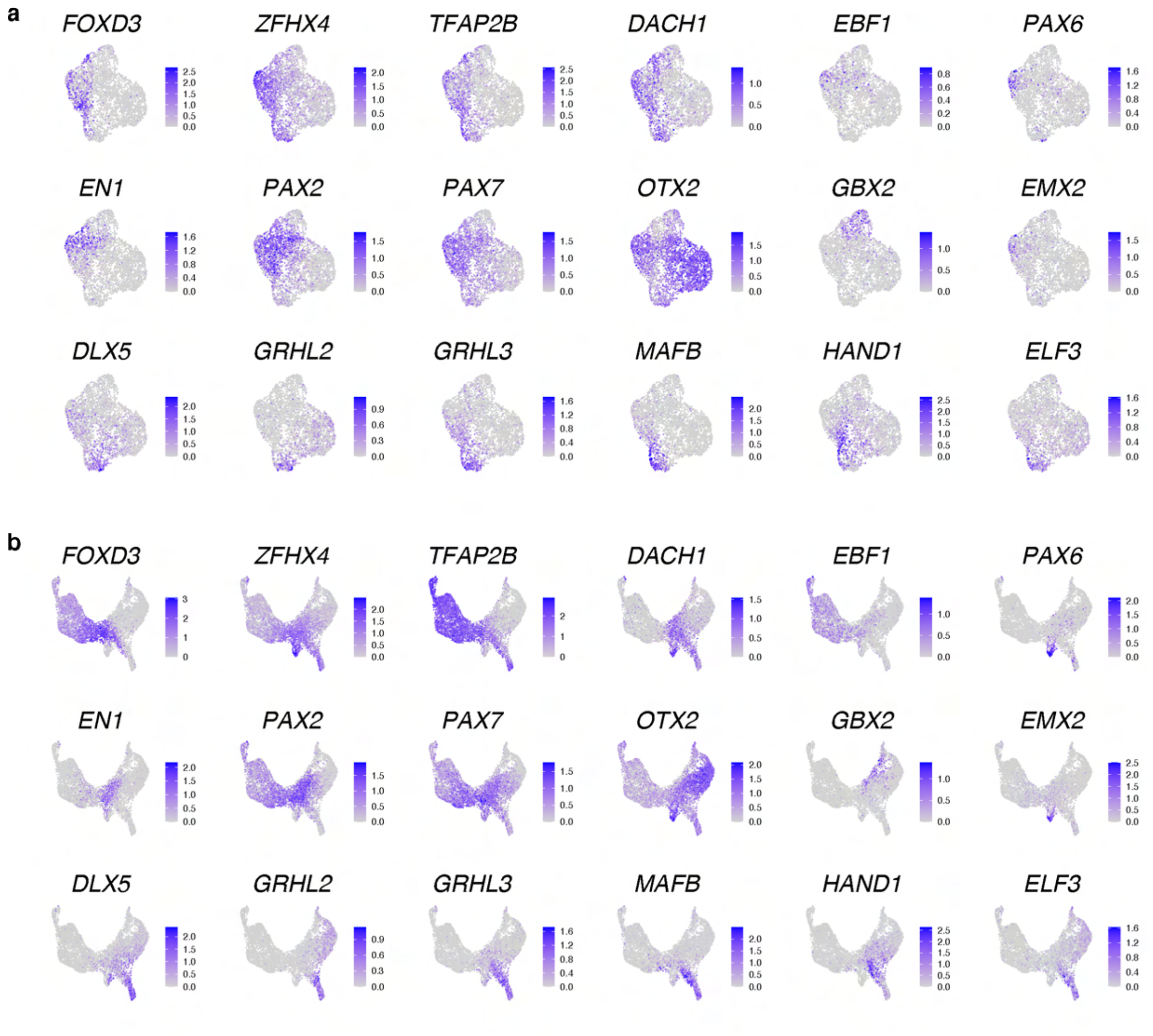

Supplementary Figure 9. A variety of genes in expressed in subsets of NPB (neural plate border)-like cells. (a) Feature plots of genes expressed in subsets of NPB-like cell clusters. (b) Feature plots of same genes plotted on original merged data.

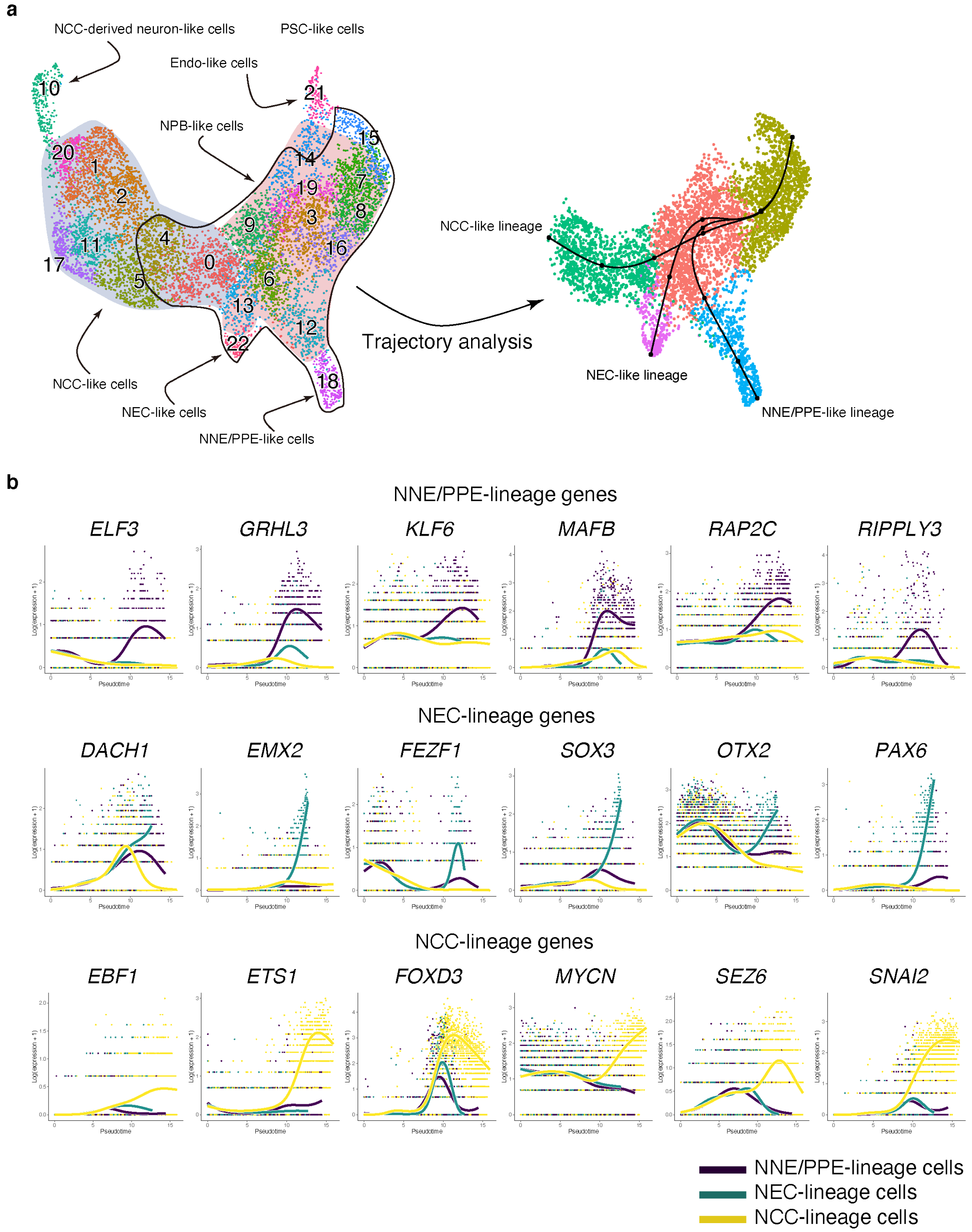
a
NCC-derived neuron-like cells
PSC-like cells
Endo-like cells
NPB-like cells
NCC-like cells
NEC-like cells
NNE/PPE-like cells
Trajectory analysis
NCC-like lineage
NEC-like lineage
NNE/PPE-like lineage
b
NNE/PPE-lineage genes
ELF3
GRHL3
KLF6
MAFB
RAP2C
RIPPLY3
NEC-lineage genes
DACH1
EMX2
FEZF1
SOX3
OTX2
PAX6
NCC-lineage genes
EBF1
ETS1
FOXD3
MYCN
SEZ6
SNAI2
NNE/PPE-lineage cells
NEC-lineage cells
NCC-lineage cells

Supplementary Figure 10. Trajectory analysis of NPB (neural plate border)-like cells.

(a) Subsets of cells including NPB-like cells and their derivatives were extracted and trajectory analysis was performed. Trajectories toward each lineage was calculated. Endo: endoderm, NCC: neural crest cell, NEC: neuroepithelial cell, NNE/PPE: non-neural ectoderm/pre-placodal epithelium, PSC: pluripotent stem cell. (b) Expression dynamics of representative genes modulated during the differentiation into each derivative of NPB-like cells.

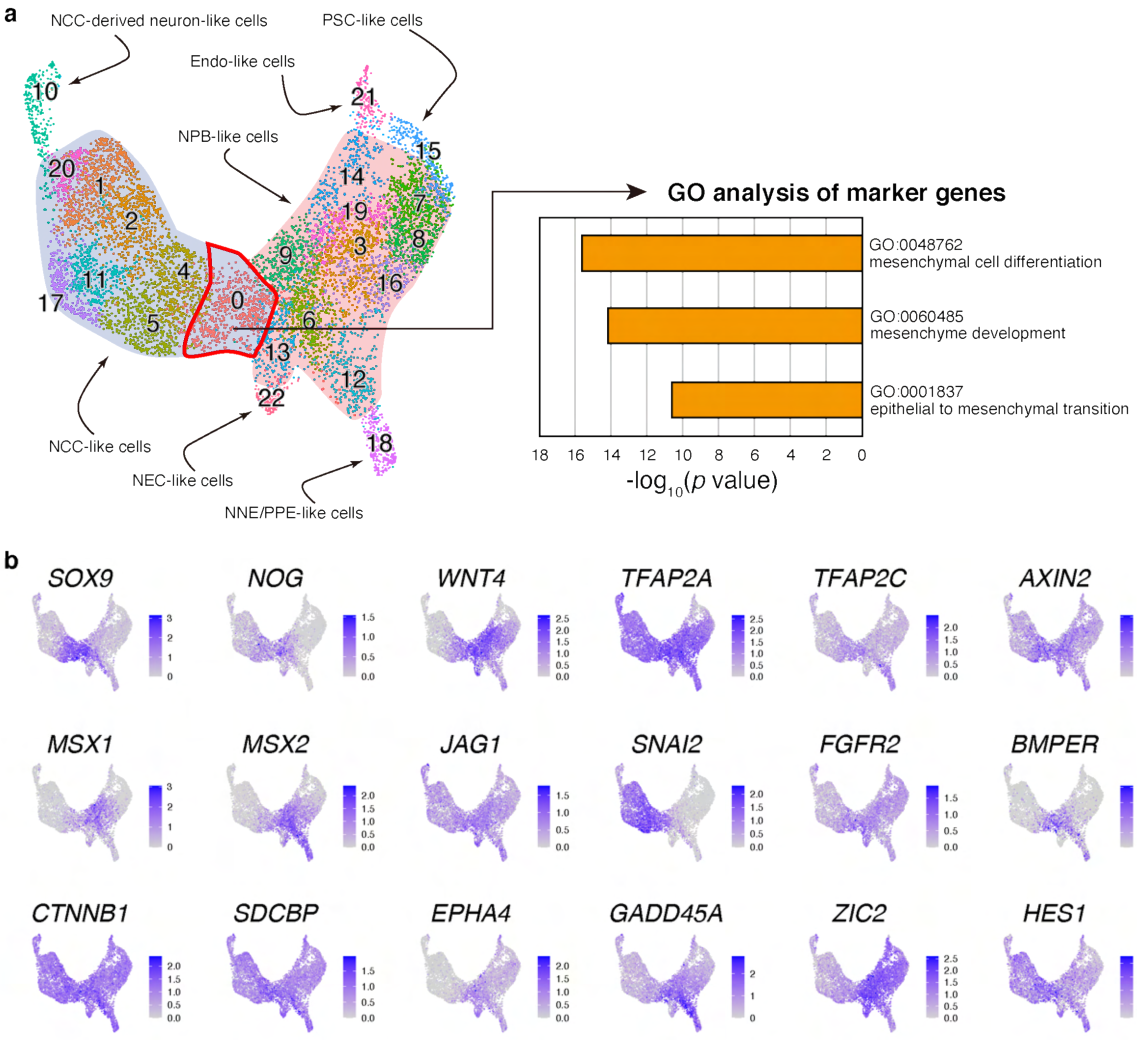

Supplementary Figure 11. Expression of epithelial-mesenchymal transition-related genes during the differentiation into NCC (neural crest cell)-like cells.

(a) Gene ontology (GO) analysis was performed on marker genes for cluster 0 of merged scRNA-seq data which included nascent NCC-like cells. The result indicated the enrichment of genes related to mesenchymal development and epithelial-mesenchymal transition. Endo: endoderm, NEC: neuroepithelial cell, NNE/PPE: non-neural ectoderm/pre-placodal epithelium, NPB: neural plate border, PSC: pluripotent stem cell. (b) Feature plots of representative genes related to mesenchymal development and epithelial-mesenchymal transition.

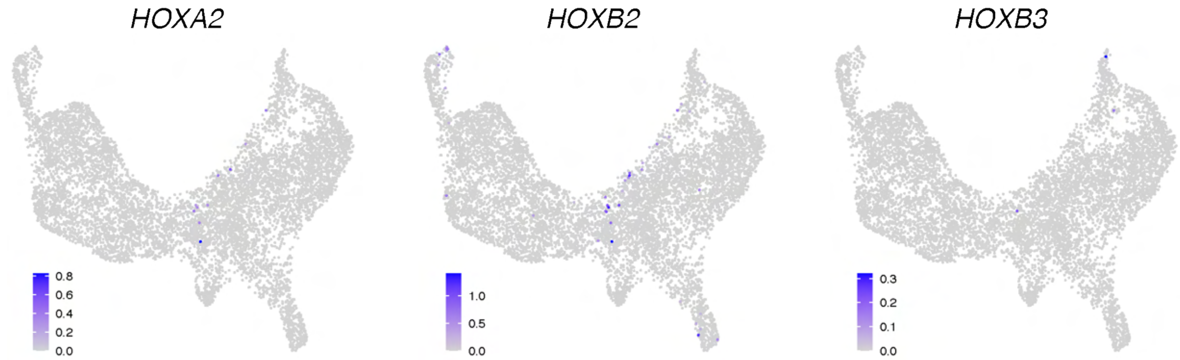

Supplementary Figure 12. Expression of HOX genes in the aggregates.

Representative feature plots of *HOX* genes on merged scRNA-seq data. *HOX*-positive cells were very rare.

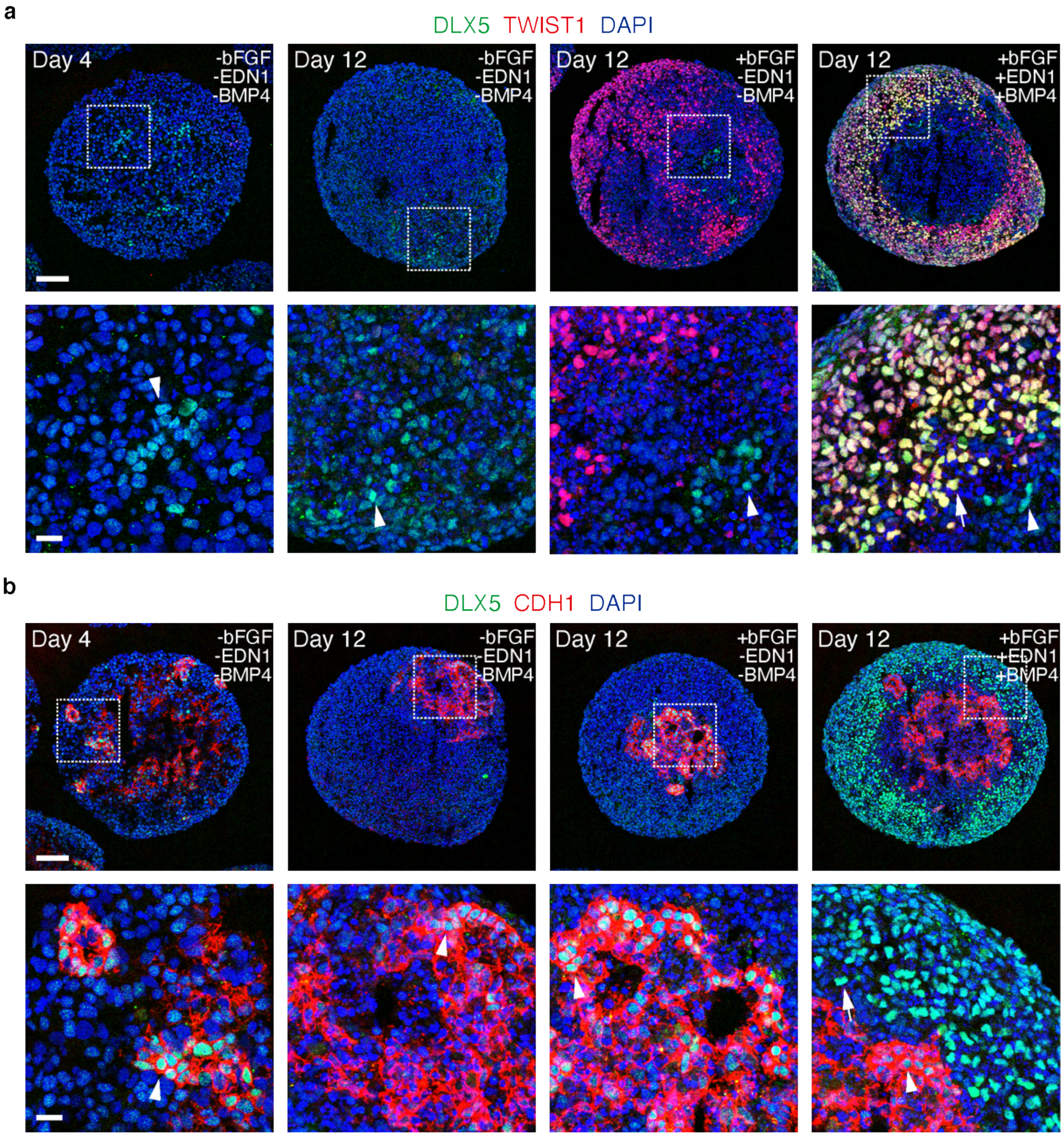
a
DLX5 TWIST1 DAPI
Day 4
-bFGF
-EDN1
-BMP4
Day 12
-bFGF
-EDN1
-BMP4
Day 12
+bFGF
-EDN1
-BMP4
Day 12
+bFGF
+EDN1
+BMP4
b
DLX5 CDH1 DAPI
Day 4
-bFGF
-EDN1
-BMP4
Day 12
-bFGF
-EDN1
-BMP4
Day 12
+bFGF
-EDN1
-BMP4
Day 12
+bFGF
+EDN1
+BMP4

Supplementary Figure 13. DLX5 expression in the aggregate at day 4 and day 12.

To evaluate the result of real-time PCR analysis on *DLX5* expression in bFGF (basic fibroblast growth factor)-treated and non-treated aggregates (Fig. 6b), immunohistochemistry was performed on the aggregates cultured in several different conditions. (a) Immunostaining of DLX5 (green) and craniofacial mesenchymal cell marker TWIST1 (red). (b) Immunostaining of DLX5 (green) and epithelial cell marker CDH1 (red). DLX5 was expressed in epithelial cells in bFGF-treated and non-treated aggregates at day 12 as same as day-4 aggregates, indicating that NCC-like cells did not express DLX5 in those conditions. As a positive control, DLX5 was expressed NCC-derived mesenchymal cells in the aggregates treated with endothelin 1(EDN1) and bone morphogenetic protein 4(BMP4) (related to Fig. 7). Scale bars, 100 μm (upper) and 20 μm(bottom) in both (a) and (b). Three independent cultures were used for experiment and representative images are shown for each figure.

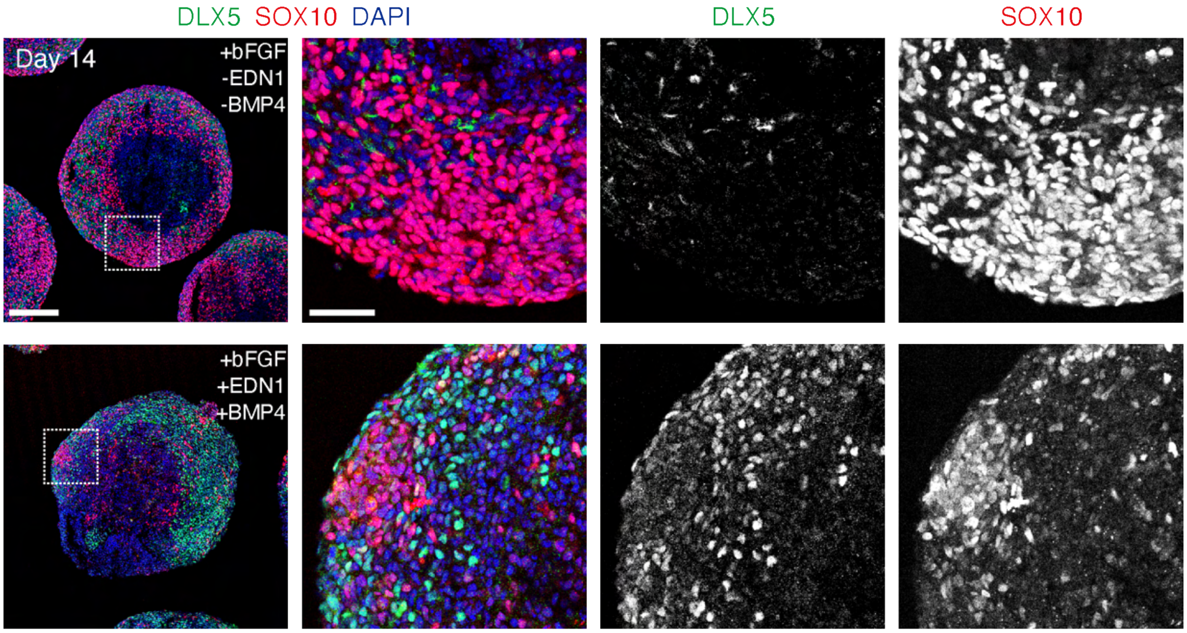

Supplementary Figure 14. Expression of DLX5 and SOX10 in day-14 aggregates.

Upper images showed immunostaining of DLX5 (green) and SOX10 (red) in bFGF (basic fibroblast growth factor)-treated aggregates, which would differentiate into MX (maxillary arch)-like state, at day 14. In this condition, most NCC (neural crest cell)-like cells in the aggregates expressed SOX10 at this stage. Bottom images were taken from EDN1/BMP4-treated aggregates, which would differentiate into MN (mandibular arch)-like state. In these aggregates, a lot of NCC-like cells expressed MN marker DLX5 at this stage but subsets of cells still expressed SOX10 not DLX5, suggesting that they had not been committed to MN lineage yet. Scale bars, 200 µm (left) and 50 µm (right). Three independent cultures were used for experiment and representative images are shown for each figure. BMP4: bone morphogenetic protein 4, EDN1: endothelin 1.

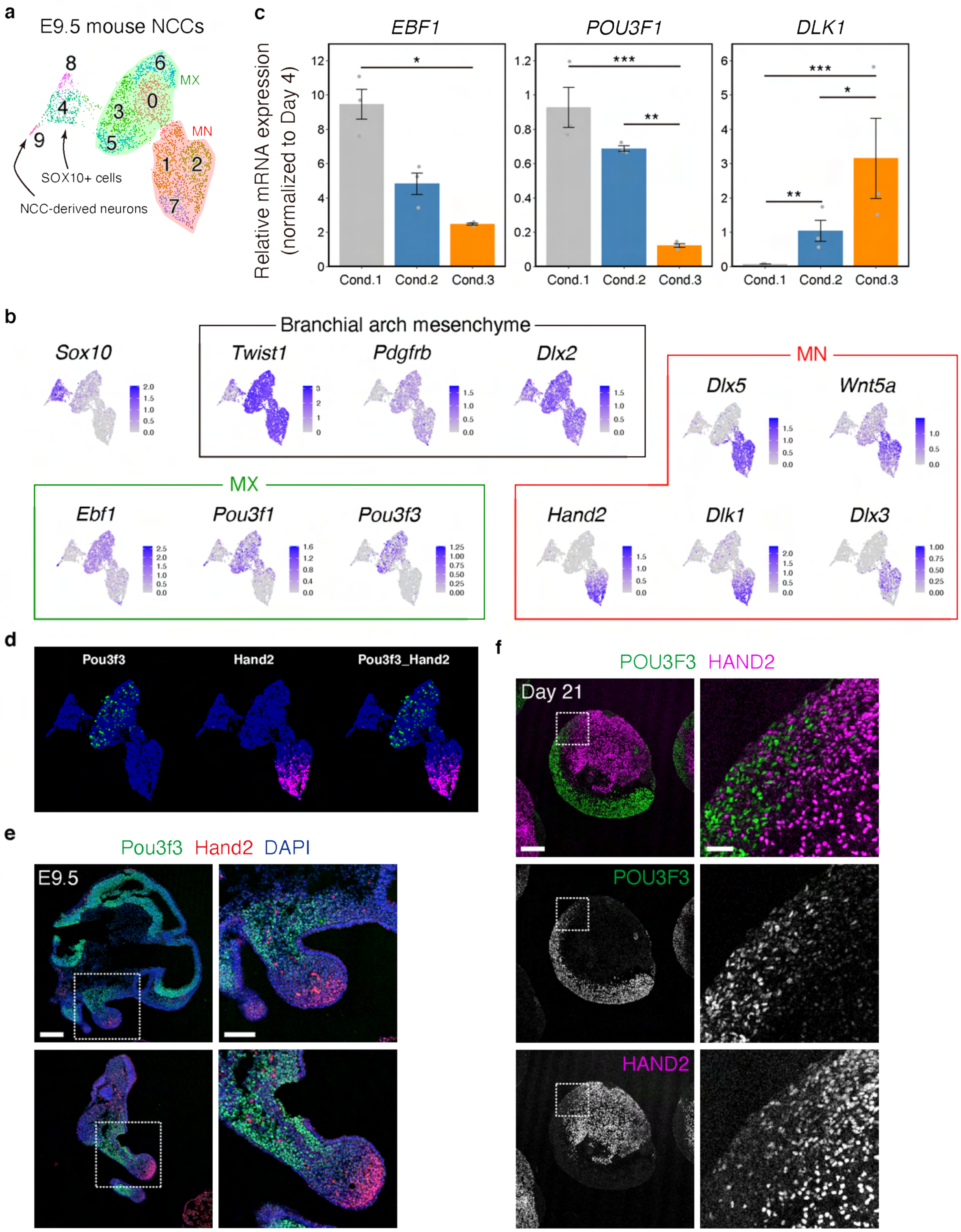
a
E9.5 mouse NCCs
MX
MN
SOX10+ cells
NCC-derived neurons
c
EBF1
POU3F1
DLK1
Relative mRNA expression (normalized to Day 4)
Cond.1
Cond.2
Cond.3
b
Sox10
Branchial arch mesenchyme
Twist1
Pdgfrb
Dlx2
MN
Dlx5
Wnt5a
MX
Ebf1
Pou3f1
Pou3f3
Hand2
Dlk1
Dlx3
d
Pou3f3
Hand2
Pou3f3_Hand2
e
Pou3f3 Hand2 DAPI
E9.5
f
POU3F3 HAND2
Day 21
POU3F3
HAND2

Supplementary Figure 15. Analysis of mouse embryonic branchial arch.

(a) Uniform manifold approximation and projection of E9.5 mouse NCCs (neural crest cells). Data were taken from reference 58 in main text. (b) Feature plots of representative markers including some presumptive markers for early MX (maxillary arch) and MN (mandibular arch). Cond.: condition. (c) Real-time PCR analysis of presumptive MX/MN markers on day-21 aggregates (related to Fig.8). Data are presented as mean ± SEM (n = 3 independent experiments). One-way ANOVA with Tukey-Kramer test was used. Source data are provided as a Source Data file. (d) Feature plots of *Pou3f3* and *Hand2* suggested segregation of their expression in mouse branchial arch at E9.5. (e) Representative images of immunostaining of Pou3f3 (green) and Hand2 (red) on head of E9.5 mouse embryos. In the first branchial arch, Pou3f3 was expressed in proximal region and Hand2 was expressed in distal region. Their expression was segregated as suggested in (d). Two sections of different mediolateral levels were represented. Three independent samplings were performed and representative images are shown. Scale bars, 200 µm (left) and 100 µm (right). (f) Magnifications of immunostaining of POU3F3 (green) and HAND2 (magenta) on a temporally-treated aggregate shown in Fig. 8b. POU3F3 and HAND2 did not co-expressed as *in vivo*. Scale bars, 200 µm (left) and 40 µm (right). Three independent cultures were used for experiment and representative images are shown.

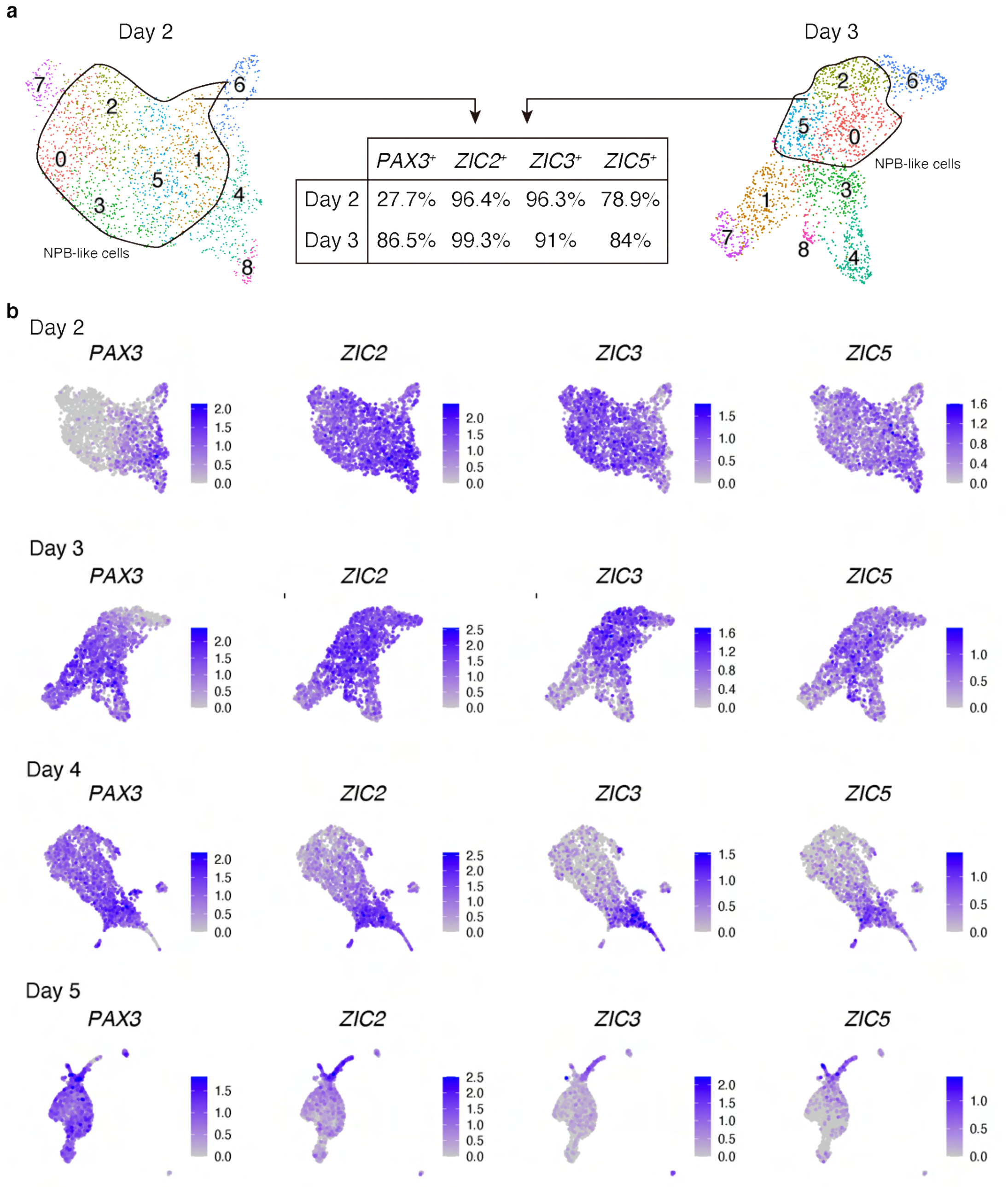

Supplementary Figure 16. Expression of *PAX3* and *ZIC* family in the aggregates.

(a) Uniform manifold approximation and projection of day-2 and day-3 aggregates. Percentage of *PAX3*$^{+}$, *ZIC2*$^{+}$, *ZIC3*$^{+}$, and *ZIC5*$^{+}$ cells in NPB (neural plate border)-like cells were shown in table. (b) Feature plots of *PAX3*, *ZIC2*, *ZIC3*, and *ZIC5*, suggesting broad expression of *ZIC* family in NPB-like cells at any developmental stage.

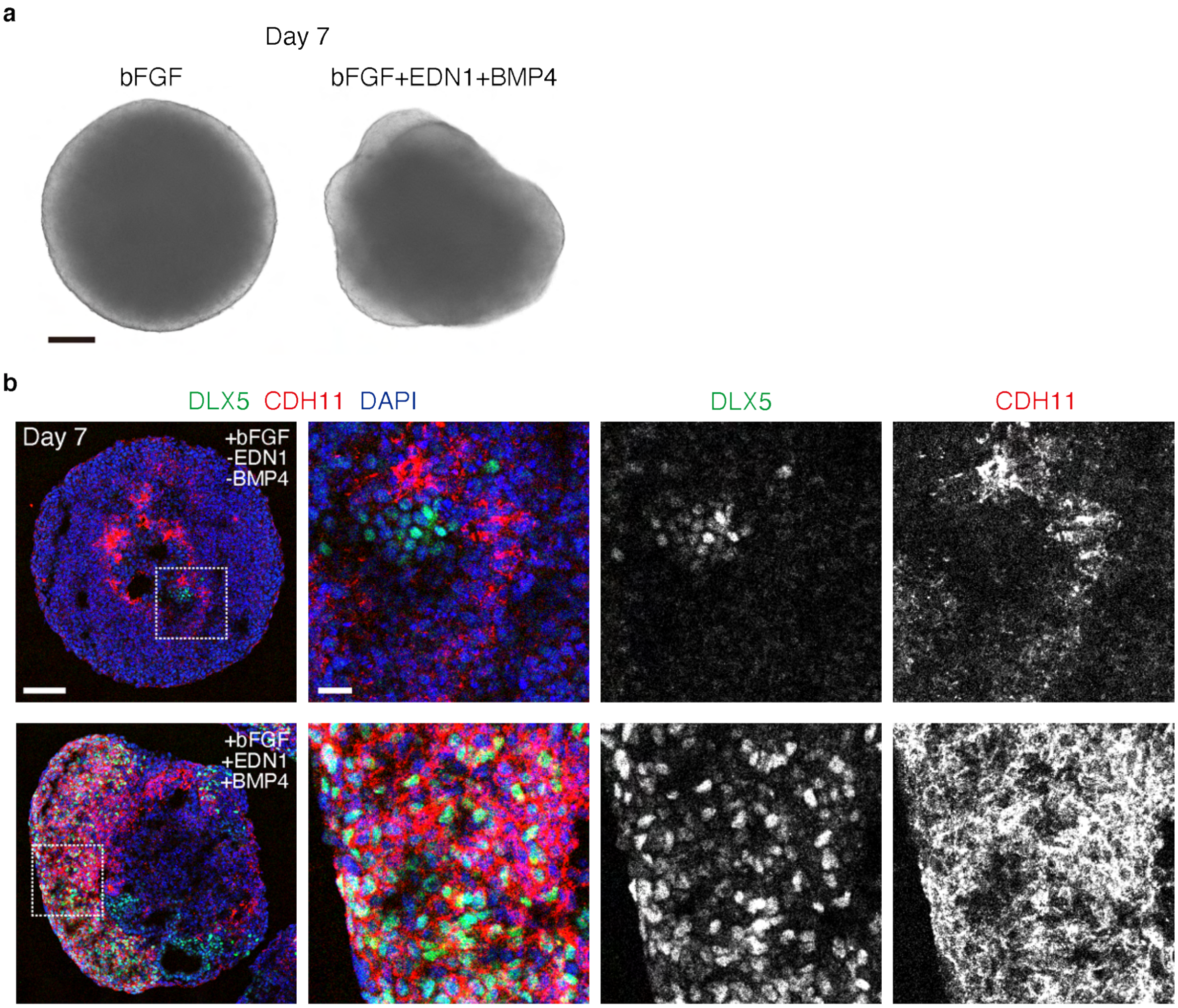
a
Day 7
bFGF
bFGF+EDN1+BMP4
b
DLX5 CDH11 DAPI
DLX5
CDH11
Day 7
+bFGF
-EDN1
-BMP4
+bFGF
+EDN1
+BMP4

Supplementary Figure 17. Expression of CDH11 in day-7 aggregates.

(a) Representative images of bFGF-treated and EDN1/BMP4 treated aggregate at day 7. EDN1/BMP4-treated aggregates exhibited biased deformation at this stage. Scale bar, 200 μm (b) Immunostaining of DLX5 (green) and CDH11 (red) on day-7 aggregates. At this stage, $DLX5^{+}/CDH11^{+}$ cells seemed to be localized in deformed regions. NCC (neural crest cell)-like cells in bFGF-treated aggregates did not seem to express CDH11. In bFGF-treated aggregates, DLX5 would be expressed in $CDH1^{+}$ cells as shown in Supplementary Fig. 13 and CDH11 was expressed in only inner region of the aggregates. Scale bars, 100 μm (left) and 20 μm (right). Three independent cultures were used for experiment and representative images are shown for each figure. bFGF: basic fibroblast growth factor, BMP4: bone morphogenetic protein 4, EDN1: endothelin 1.

Supplementary table 1. Primary antibodies used for immunohistochemistry

Target	Vendor	Catalog No.	Host	Dilution
BARX1	Atlas Antibodies	HPA055858	Rabbit	1:1000
CDH1	Takara	M108	Rat	1:1000
CDH6	DSHB	CCD6B-1	Mouse	1:1000
CDH11	Cell Signaling Technology	13577	Mouse	1:500
DLX2	Bio Academia	74-116	Guinea pig	1:3000
DLX5	Atlas antibodies	HPA005670	Rabbit	1:5000
EBF2	R&D Systems	AF7006	Sheep	1:200
FOXD3	Custom made	-	Rabbit	1:250
GSC	R&D Systems	AF4086	Goat	1:2000
HAND2	Santa Cruz	sc-9409	Goat	1:500
ISL1/2	DSHB	39.4D5	Mouse	1:250
LHX6	Santa Cruz	sc-271433	Mouse	1:200
MITF	Exalpha	X2398M	Mouse	1:500
NKX3.2	Atlas Antibodies	HPA027564	Rabbit	1:200
p75	Promega	G323A	Rabbit	1:150
PAX6	BD Pharmingen	561462	Mouse	1:1000
PAX7	R&D Systems	MAB-1675	Mouse	1:1000
POU3F3	Atlas Antibodies	HPA067151	Rabbit	1:1000
POU5F1	BD Transduction Laboratories	611203	Mouse	1:500
PRRX1	Abcam	ab211292	Rabbit	1:2000
RUNX2	MBL	D130-3	Mouse	1:1000
SIX1	Sigma Aldrich	HPA001893	Rabbit	1:1000
SMA	DAKO	M0851	Mouse	1:100
SOX2	Santa Cruz	sc-17320	Goat	1:200
SOX9	Santa Cruz	Sc-166505	Mouse	1:200
SOX10	R&D Systems	AF2864	Goat	1:1000
SOX17	R&D Systems	AF1924	Goat	1:200
SP7	Abcam	209484	Rabbit	1:1000
TUJ1	Covance	MMS-435P	Mouse	1:500
TWIST1	Santa Cruz	sc-81417	Mouse	1:200

Supplementary table 2. Secondary antibodies used for immunohistochemistry

Target	Conjugate	Vendor	Catalog No.	Host	Dilution
Guinea pig	Alexa Fluor 488	Jackson ImmunoResearch Labs	706-545-148	Donkey	1:1000
Guinea pig	Cyanine Cy5	Jackson ImmunoResearch Labs	706-175-148	Donkey	1:1000
Goat	Alexa Fluor 488	Jackson ImmunoResearch Labs	705-545-003	Donkey	1:1000
Goat	Cyanine Cy3	Jackson ImmunoResearch Labs	705-165-003	Donkey	1:1000
Goat	Cyanine Cy5	Jackson ImmunoResearch Labs	705-175-003	Donkey	1:1000
Mouse	Alexa Fluor 488	Jackson ImmunoResearch Labs	715-545-150	Donkey	1:1000
Mouse	Cyanine Cy3	Jackson ImmunoResearch Labs	715-165-150	Donkey	1:1000
Mouse	Cyanine Cy5	Jackson ImmunoResearch Labs	715-175-150	Donkey	1:1000
Rat	Alexa Fluor 488	Jackson ImmunoResearch Labs	712-545-150	Donkey	1:1000
Rat	Cyanine Cy3	Jackson ImmunoResearch Labs	712-165-150	Donkey	1:1000
Rat	Cyanine Cy5	Jackson ImmunoResearch Labs	712-175-150	Donkey	1:1000
Rabbit	Alexa Fluor 488	Jackson ImmunoResearch Labs	711-545-152	Donkey	1:1000
Rabbit	Cyanine Cy3	Jackson ImmunoResearch Labs	711-165-152	Donkey	1:1000
Rabbit	Cyanine Cy5	Jackson ImmunoResearch Labs	711-175-152	Donkey	1:1000
Sheep	Alexa Fluor 488	Jackson ImmunoResearch Labs	713-545-003	Donkey	1:1000

Supplementary table 3. Antibodies used for flow cytometry

Target	Vendor	Catalog No.	Host	Dilution
Isotype control	Miltenyi Biotec	130-120-709	human	1:50
p75-APC	Miltenyi Biotec	130-112-602	human	1:50

Supplementary table 4. Primer sequences for real-time PCR analysis

Target	Forward	Reverse
AXIN2	GAGTGGACTTGTGCCGACTTCA	GGTGGCTGGTGCAAAGACATAG
BARX1	TCCACGCCGGACAGAATAGA	AGTAAGCTGCTCGCTCGTTG
BMP2	ACGCTCTTTCAATGGACGTG	GGAAGCAGCAACGCTAGAAG
BMP4	CTTTACCGGCTTCAGTCTGG	GGGATGCTGCTGAGGTTAAA
CDH1	GGATGTGCTGGATGTGAATG	CTCAAAATCCTCCCTGTCCA
DLK1	GCGAGGATGACAATGTTTG	AGCAGGCCCGAACATCTCT
DLX2	CATGGGTTCCTACCAGTACCAA	CCGAATTTCAGGCTCAAGGTC

Supplementary table 4. Primer sequences for real-time PCR analysis (continued)

**Target**	**Forward**	**Reverse**
DLX3	TACCTACGGAGCCTCCTACC	CTTTCGGACCTTCTTGGGCT
DLX5	GCACATGGGTTCCTACCAGT	ACTTTCTTTGGCTTCCCGTT
EBF1	CCCTCTTATCTGGAACATGCTACT	ACGGATGGCATGAGGAGTG
EYA1	CAGGCACCATACAGCTACCA	AAGACGGATAGTCCTGCTGTG
FOXD3	GCATCTGCGAGTTCATCAGC	CGTTGAGTGAGAGGTTGTGG
FRZB	AGCCCTGTAAGTCTGTGTGC	ACGAGTGGCGGTACTTGATG
GAPDH	CCCATCACCATCTTCCAGGAG	CTTCTCCATGGTGGTGAAGACG
GSC	TCTCAACCAGCTGCACTGTC	GGCGGTTCTTAAACCAGACC
HAND2	GACCGACGTGAAAGAGGAGA	TTTCTTGTCGTTGCTGCTCAC
LHX6	GCAGAACAGCTGCTACATCAAGAA	CAGTCGCTGGCGTAGATCTGTC
MSX2	CTCTCCACGAAGGCAGTACC	GCCTCCGCCTACAGAACAAA
NANOG	TGGACACTGGCTGAATCCTTC	CGTTGATTAGGCTCCAACCAT
NKX3.2	GCCGCTTCCAAAGACCTAGAG	CCAACACCGTCGTCCTCG
PAX6	CACCTACAGCGCTCTGCCGC	CCCGAGGTGCCCATTGGCTG
PAX7	TGACAGCTTCATGAATCCGG	GATGGAGAAGTCAGCCTGTG
POU3F1	GCTCGAGAGCCACTTTCTCA	GGCGCGTATACATCGTCCAT
POU3F3	TGGACTCAACAGCCACGAC	CTTGAACTGCTTGGCGAAC
PRRX1	CTGATGCTTTTGTGCGAGAA	ACTTGGCTCTTCGGTTCTGA
SOX10	CTCTGGAGGCTGCTGAA	TGGGCTGGTACTTGTAGTC
TWIST1	TCCATTTTCTCCTTCTCTGGAA	GTCCGCGTCCCACTAGC
WNT1	CTCATGAACCTTCACAACAACGA	ATCCCGTGGCACTTGCA
WNT4	CTAGCCCCGACTTCTGTGAG	TTGGACGTCTTGTTGCATGT
WNT5A	CGCCCAGGTTGTAATTGAAG	GCATGTGGTCCTGATACAAAGT
WNT7B	GCAGGAAGGTTCTAGAGG	GTTGTACTTCTCCTTCAGC
