# Supplementary material for: In vitro induction of patterned branchial arch-like aggregate from human pluripotent stem cells
Source: Nat Commun. 2024 Feb 14;15:1351. doi: 10.1038/s41467-024-45285-0 (PMC10867012; doi:10.1038/s41467-024-45285-0)
Supplement: Supplementary file 3 — Reporting Summary [file 41467_2024_45285_MOESM3_ESM.pdf]

nature portfolio

Corresponding author(s): Yusuke Seto and Mototsugu Eiraku

Last updated by author(s): Jan 10, 2024

Reporting Summary

Nature Portfolio wishes to improve the reproducibility of the work that we publish. This form provides structure for consistency and transparency in reporting. For further information on Nature Portfolio policies, see our Editorial Policies and the Editorial Policy Checklist.

Statistics

For all statistical analyses, confirm that the following items are present in the figure legend, table legend, main text, or Methods section.

n/a	Confirmed	
☐	☒	The exact sample size (*n*) for each experimental group/condition, given as a discrete number and unit of measurement
☐	☒	A statement on whether measurements were taken from distinct samples or whether the same sample was measured repeatedly
☐	☒	The statistical test(s) used AND whether they are one- or two-sided<br>*Only common tests should be described solely by name; describe more complex techniques in the Methods section.*
☒	☐	A description of all covariates tested
☐	☒	A description of any assumptions or corrections, such as tests of normality and adjustment for multiple comparisons
☐	☒	A full description of the statistical parameters including central tendency (e.g. means) or other basic estimates (e.g. regression coefficient) AND variation (e.g. standard deviation) or associated estimates of uncertainty (e.g. confidence intervals)
☐	☒	For null hypothesis testing, the test statistic (e.g. *F*, *t*, *r*) with confidence intervals, effect sizes, degrees of freedom and *P* value noted<br>*Give P values as exact values whenever suitable.*
☒	☐	For Bayesian analysis, information on the choice of priors and Markov chain Monte Carlo settings
☒	☐	For hierarchical and complex designs, identification of the appropriate level for tests and full reporting of outcomes
☒	☐	Estimates of effect sizes (e.g. Cohen's *d*, Pearson's *r*), indicating how they were calculated

Our web collection on statistics for biologists contains articles on many of the points above.

Software and code

Policy information about availability of computer code

Data collection	For imaging with a TCS SP8 cofocal microscope: Leica Application Suite X (Version 3.5.5.19976)<br>For bright field imaging with a BZ-X710 microscope: BZ-X Viewer (Version 1.4.0.1)<br>For real-time PCR: QuantStudio Real-Time PCR Software 'Version 1.3)<br>For flow cytometry with a FACSAlia IIIu FACS: BD FACSDiva Software (Version 6.1.3)
Data analysis	ImageJ (1.53k). FlowJo (Version 10.8.1). Microsoft Excel for Mac (Version 16.44). R (Version 4.3.1). Packages for R: dplyr (Version 1.1.3), ggplot2 (Version 3.4.3), scCustomize (version 1.1.3), Seurat (Version 4.3.0.1), slingshot (Version2.8.0), stats (Version 4.3.1), tidyr (version 1.3.0), tradeSeq (Version 1.1.4), monocle3 (Version 1.3.3), Velocyto (Version 0.6). Python (Version 3.9.5). Packages for Python: anndata (Version 0.7.8), numpy (Version 1.20.3), pandas (Version 1.4.1), scanpy (Version 1.8.2), scipy (Version 1.8.0), scvelo (Version 0.2.4). Cell Ranger (Version 5.0.1).

For manuscripts utilizing custom algorithms or software that are central to the research but not yet described in published literature, software must be made available to editors and reviewers. We strongly encourage code deposition in a community repository (e.g. GitHub). See the Nature Portfolio guidelines for submitting code & software for further information.

Data

Policy information about availability of data

All manuscripts must include a data availability statement. This statement should provide the following information, where applicable:

- Accession codes, unique identifiers, or web links for publicly available datasets
- A description of any restrictions on data availability
- For clinical datasets or third party data, please ensure that the statement adheres to our policy

The accession number of the scRNA-seq data reported in this study is Gene Expression Omnibus (GEO): GSE199158.

Research involving human participants, their data, or biological material

Policy information about studies with human participants or human data. See also policy information about sex, gender (identity/presentation), and sexual orientation and race, ethnicity and racism.

Reporting on sex and gender	N/A
Reporting on race, ethnicity, or other socially relevant groupings	N/A
Population characteristics	N/A.
Recruitment	N/A.
Ethics oversight	N/A

Note that full information on the approval of the study protocol must also be provided in the manuscript.

Field-specific reporting

Please select the one below that is the best fit for your research. If you are not sure, read the appropriate sections before making your selection.

☒ Life sciences ☐ Behavioural & social sciences ☐ Ecological, evolutionary & environmental sciences

For a reference copy of the document with all sections, see nature.com/documents/nr-reporting-summary-flat.pdf

Life sciences study design

All studies must disclose on these points even when the disclosure is negative.

Sample size	For each experiment, at least three independent biological replicates were used.
Data exclusions	No data were excluded.
Replication	For immunohistochemistry and bright field imaging, we performed at least three independent experiments and representative images were showed in figures. For real-time PCR analysis, we performed at least three biological independent experiments and the number of biological replication are mentioned in figure legends. For flow cytometry analysis, we performed at least three biological independent experiments and the number of biological replication are mentioned in figure legend. For single cell RNA-seq, we performed single experiment and all data were collected from 32 aggregates.
Randomization	For each sample, aggregates were collected from an>96 aggregates randomly which have been cultured in V-bottom 96-well plates.
Blinding	Culture of aggregates was performed in a non-blinded manner. Image acquisition and flow cytometry analysis was performed with consistent parameters.

Reporting for specific materials, systems and methods

We require information from authors about some types of materials, experimental systems and methods used in many studies. Here, indicate whether each material, system or method listed is relevant to your study. If you are not sure if a list item applies to your research, read the appropriate section before selecting a response.

Materials & experimental systems

n/a	Involved in the study
☐	☒ Antibodies
☐	☒ Eukaryotic cell lines
☒	☐ Palaeontology and archaeology
☐	☒ Animals and other organisms
☒	☐ Clinical data
☒	☐ Dual use research of concern
☒	☐ Plants

Methods

n/a	Involved in the study
☒	☐ ChIP-seq
☐	☒ Flow cytometry
☒	☐ MRI-based neuroimaging

Antibodies

Antibodies used

For immunohistochemistry:
BARX1, Atlas Antibodies, HPA055858
CDH1, Takara, M108
CDH6, DSHB, CCD6B-1
CDH11, Cell Signaling Technology, 13577
DLX2, Bio Academia, 74-116
DLX5, Atlas antibodies, HPA005670
EBF2, R&D Systems, AF7006
FOXD3, Custom made
GSC, R&D Systems, AF4086
HAND2, Santa Cruz, sc-9409
ISL1/2, DSHB, 39.4D5
LHX6, Santa Cruz, sc-271433
MITF, Exalpha, X2398M
MSX1/2, DSHB, 4G1
NKX3.2, Atlas Antibodies, HPA027564
p75, Promega, G323A
PAX6, BD Pharmingen, 561462
PAX7, R&D Systems, MAB-1675
POU3F3, Atlas Antibodies, HPA067151
POU5F1, BD Transduction Laboratories, 611203
PRRX1, Abcam, ab211292
RUNX2, MBL, D130-3
SIX1, Sigma Aldrich, HPA001893
SMA, DAKO, M0851
SNAI2, Cell Signaling Technology, 9585
SOX2, Santa Cruz, sc-17320
SOX10, R&D Systems, AF2864
SOX17, R&D Systems, AF1924
SP7, Abcam, 209484
TUJ1, Covance, MMS-435P
TWIST1, Santa Cruz, sc-81417
Alexa Fluor 488-Donkey anti-Guinea pig IgG secondary antibody, Jackson ImmunoResearch Labs, 706-545-148
Cyanine Cy5-Donkey anti-Guinea pig IgG secondary antibody, Jackson ImmunoResearch Labs, 706-175-148
Alexa Fluor 488-Donkey anti-Goat IgG secondary antibody, Jackson ImmunoResearch Labs, 705-545-003
Cyanine Cy3-Donkey anti-Goat IgG secondary antibody, Jackson ImmunoResearch Labs, 705-165-003
Cyanine Cy5-Donkey anti-Goat IgG secondary antibody, Jackson ImmunoResearch Labs, 705-175-003
Alexa Fluor 488-Donkey anti-Mouse IgG secondary antibody, Jackson ImmunoResearch Labs, 715-545-150
Cyanine Cy3-Donkey anti-Mouse IgG secondary antibody, Jackson ImmunoResearch Labs, 715-165-150
Cyanine Cy5-Donkey anti-Mouse IgG secondary antibody, Jackson ImmunoResearch Labs, 715-175-150
Alexa Fluor 488-Donkey anti-Rat IgG secondary antibody, Jackson ImmunoResearch Labs, 712-545-150
Cyanine Cy3-Donkey anti-Rat IgG secondary antibody, Jackson ImmunoResearch Labs, 712-165-150
Cyanine Cy5-Donkey anti-Rat IgG secondary antibody, Jackson ImmunoResearch Labs, 712-175-150
Alexa Fluor 488-Donkey anti-Rabbit IgG secondary antibody, Jackson ImmunoResearch Labs, 711-545-152
Cyanine Cy3-Donkey anti-Rabbit IgG secondary antibody, Jackson ImmunoResearch Labs, 711-165-152
Cyanine Cy5-Donkey anti-Rabbit IgG secondary antibody, Jackson ImmunoResearch Labs, 711-175-152
Alexa Fluor 488-Donkey anti-Sheep IgG secondary antibody, Jackson ImmunoResearch Labs, 713-545-003

For flow cytometry:
control antibody conjugated with APC for flow cytometry, Miltenyi Biotec, 130-120-709
p75 antibody conjugated with APC for flow cytometry, Miltenyi Biotec, 130-112602

Validation

BARX1, Atlas Antibodies, HPA055858: validated by the manufacture.

CDH1, Takara, M108: validated by the manufacture.
CDH6, DSHB, CCD6B-1: Nakagawa S and Takeichi M, Development, 1998.
CDH11, Cell Signaling Technology, 13577: validated by the manufacture.
DLX2, Bio Academia, 74-116: validated by the manufacture.
DLX5, Atlas antibodies, HPA005670: validated by the manufacture.
EBF2, R&D Systems, AF7006: Su S, et al., Cell Reports, 2018.
FOXD3, Custom made: Rabbit polyclonal antibody was raised against recombinant fragment of mouse FOXD3 protein (232aa-465aa) which is 86% identical to 243aa-459aa of human FOXD3. Specificity of antibody was validated by immunohistochemistry on mouse embryonic tissues.
GSC, R&D Systems, AF4086: validated by the manufacture.
HAND2, Santa Cruz, sc-9409: Huyen DV. and Bany BM., Reproduction, 2011.
ISL1/2, DSHB, 39.4D5: Bu L, et al., Nature, 2009.
LHX6, Santa Cruz, sc-271433: The manufacture recommends this antibody for detecting human LHX6. Specificity of this antibody was validated by immunohistochemistry on mouse embryonic tissues (For example, Lozovaya N., et al., Nature Communications, 2018.)
MITF, Exalpha, X2398M: Kuwahara A., et al., Nature Communications, 2015.
NKX3.2, Atlas Antibodies, HPA027564: validated by the manufacture.
p75, Promega, G323A: This antibody was raised against human p75. Specificity of this antibody was validated by immunohistochemistry on mouse embryonic tissues (For example, Chandler RL. and Magnuson T., Developmental Biology, 2016.)
PAX6, BD Pharmingen, 561462: validated by the manufacture.
PAX7, R&D Systems, MAB-1675: validated by the manufacture.
POU3F3, Atlas Antibodies, HPA067151: validated by the manufacture.
POU5F1, BD Transduction Laboratories, 611203: validated by the manufacture.
PRRX1, Abcam, ab211292: validated by the manufacture.
RUNX2, MBL, D130-3: Ali SA. et al., Journal of Cell Science, 2012.
SIX1, Sigma Aldrich, HPA001893: validated by the manufacture.
SMA, DAKO, M0851: Piersma B., et al., The Americal Journal of Pathology, 2015.
SOX2, Santa Cruz, sc-17320: Liu GH., et al., Nature, 2011.
SOX10, R&D Systems, AF2864: validated by the manufacture.
SOX17, R&D Systems, AF1924: validated by the manufacture.
SP7, Abcam, 209484: validated by the manufacture.
TUJ1, Covance, MMS-435P: validated by the manufacture.
TWIST1, Santa Cruz, sc-81417: Zhao B., et al., Cellulaar Oncology, 2023.
p75 antibody conjugated with APC for flow cytometry, Miltenyi Biotec, 130-112602: validated by the manufacture.

Eukaryotic cell lines

Policy information about cell lines and Sex and Gender in Research

Cell line source(s)	KhES-1, KthES11 and 253G1 cell lines were established in Kyoto University.<br>Sex of KhES-1, KthES11, 253G1 pluripotent stem cell lines are female.
Authentication	KhES-1: https://cellbank.brc.riken.jp/cell_bank/CellInfo/?cellNo=HES0001&lang=En<br>KthES11: https://www.sciencedirect.com/science/article/pii/S1873506121002294<br>253G1: https://cellbank.brc.riken.jp/cell_bank/CellInfo/?cellNo=HPS0002&lang=En
Mycoplasma contamination	The cell lines were not tested for mycoplasma contamination.
Commonly misidentified lines<br>(See ICLAC register)	We did not use commonly misidentified lines.

Animals and other research organisms

Policy information about studies involving animals; ARRIVE guidelines recommended for reporting animal research, and Sex and Gender in Research

Laboratory animals	Pregnant mice (Slc:ICR, 2-3 month old) were purchased from SHIMIZU Laboratory Supplies Co.
Wild animals	We did not use wild animals.
Reporting on sex	We did not consider sex of embryos in this study.
Field-collected samples	There were no field-collected samples.
Ethics oversight	All animal procedures comply with all relevant ethical regulations and guideline for animal studies approved by the Research Ethical Committee of Kyoto University.

Note that full information on the approval of the study protocol must also be provided in the manuscript.

Plants

Seed stocks | *Report on the source of all seed stocks or other plant material used. If applicable, state the seed stock centre and catalogue number. If plant specimens were collected from the field, describe the collection location, date and sampling procedures.*

Novel plant genotypes | *Describe the methods by which all novel plant genotypes were produced. This includes those generated by transgenic approaches, gene editing, chemical/radiation-based mutagenesis and hybridization. For transgenic lines, describe the transformation method, the number of independent lines analyzed and the generation upon which experiments were performed. For gene-edited lines, describe the editor used, the endogenous sequence targeted for editing, the targeting guide RNA sequence (if applicable) and how the editor was applied.*

Authentication | *Describe any authentication procedures for each seed stock used or novel genotype generated. Describe any experiments used to assess the effect of a mutation and, where applicable, how potential secondary effects (e.g. second site T-DNA insertions, mosiacism, off-target gene editing) were examined.*

Flow Cytometry

Plots

Confirm that:

☒ The axis labels state the marker and fluorochrome used (e.g. CD4-FITC).

☒ The axis scales are clearly visible. Include numbers along axes only for bottom left plot of group (a 'group' is an analysis of identical markers).

☐ All plots are contour plots with outliers or pseudocolor plots.

☒ A numerical value for number of cells or percentage (with statistics) is provided.

Methodology

Sample preparation | The aggregates were pooled and dissociated using TrypLE Express (Gibco) at 37 °C for 8 min. The reaction was stopped by adding PBS, and the cells were filtered through a cell strainer. Dissociated cells were stained with an allophycocyanin (APC)-conjugated anti-p75 antibody (1:50; Miltenyi Biotec) or APC-conjugated isotype control antibody (1:50; Miltenyi Biotec) according to the manufacturer's instructions.

Instrument | A FACSAria IIIu flow cytometer (Bectin Dickinson) was used.

Software | For data collection, FACSDiva software (Becton Dickinson) was used. For data analysis, FLOW Jo software was used.

Cell population abundance | We did not perform sorting experiments.

Gating strategy | Forward scatter and side scatter were used to remove the dead cells and debris. Then the intensity of APC was measured. APC-conjugated isotype control antibody was used for negative control.

☒ Tick this box to confirm that a figure exemplifying the gating strategy is provided in the Supplementary Information.
